# Comment on ‘hemoglobin A1c and serum glucose levels and risk of gastric cancer: A systematic review and meta-analysis’

**DOI:** 10.3389/fendo.2022.966464

**Published:** 2022-10-07

**Authors:** Haiyang Wang, Yamei Wang, Huawen Xia

**Affiliations:** Department of Interventional Surgery, Handan First Hospital, Handan, China

**Keywords:** hemoglobin A1c (HbA1c), serum glucose, gastric cancer, systematic review, meta-analysis

In a recent systematic review and meta-analysis, Zheng et al. (1) examined associations between serum levels of hemoglobin A1c (HbA1c) or glucose and the risk of gastric cancer. They included 12 studies and concluded that serum HbA1c levels >6% were associated with an increased risk of gastric cancer (HR1.36,95%CI 1.06-1.74). The finding is of major significance. There are, however, a few concerns with the current study that should be discussed.

The study showed that there was moderate heterogeneity across the studies about associations between HbA1c and the risk of gastric cancer. From the Figure 1 of the study, we can see that among the 5 studies included, only one of them, the Ikeda’s study (from Japan), showed that HbA1c levels >6% increased the risk of gastric cancer, while the other four studies showed no associations between the elevated HbA1c and the risk of gastric cancer. We conducted a sensitivity analysis and found that after excluding Ikeda’s study, heterogeneity across the studies decreased to 0, that was, inter-study heterogeneity disappeared completely, as shown in **Figure 1**. The forest plot showed that elevated HbA1c levels did not increase the risk of gastric cancer, which was different from the authors’ final conclusion. Therefore, we considered Ikeda’s study as the main source of heterogeneity.

**Figure 1:**
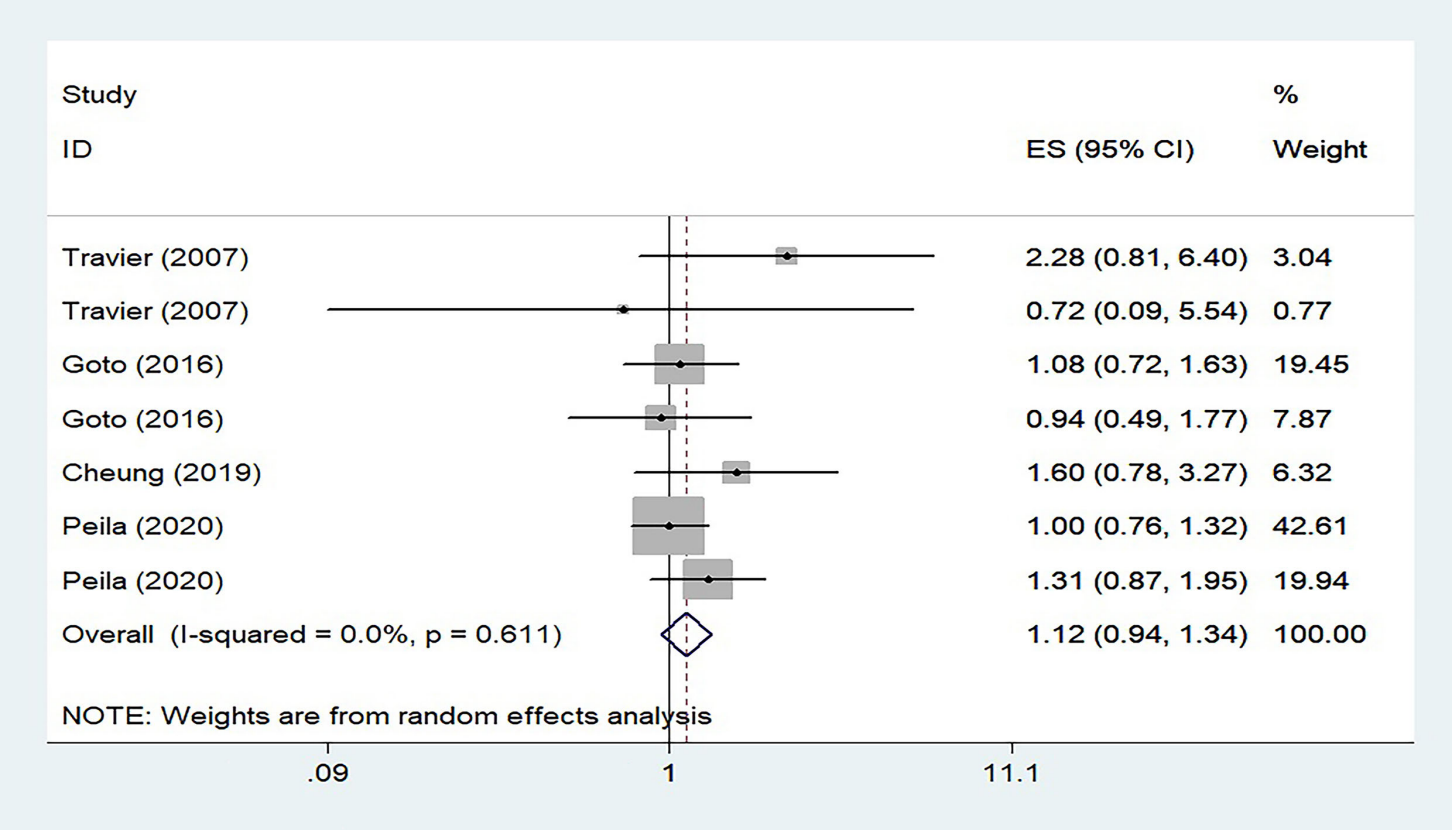
Forest plot of risk estimates for associations between serum HbA1c levels and risk of gastric cancer after eliminating Ikeda’s study.

We tried to find the differences between Ikeda’s study and other studies and found that this study had the smallest sample size, with 427 patients with HbA1c at 6.0-6.9% and only 101 patients with HbA1c ≥7%. As we all know, Japan is a country with a high incidence of gastric cancer in the world, and the proportion of people with gastric cancer will undoubtedly be higher than other studies (2, 3). Meanwhile, the sample size of the population included in Ikeda’s study is small, which cannot represent the whole specific population. Compared with the study with larger sample size, it is more likely to be interfered by confounding factors, leading to HR deviation. In another study from Japan, Goto included 29, 629 people and found that elevated HbA1c levels did not increase the risk of gastric cancer (4). Therefore, we consider that the different conclusions among studies may be caused by the different sample size.

Our reconstructed forest plot, based on the data provided by the authors in the paper, showed that elevated HbA1c levels did not increase the risk of gastric cancer, which was different from the authors’ final conclusion. In summary, more evidence from large prospective studies is still needed to confirm the association between elevated level of HbA1c and risk of gastric cancer.

## Author contributions

YW, HW, and HX wrote the letter. All authors contributed to the article and approved the submitted version.

## Acknowledgments

The authors would like to thank the participating members.

## Conflict of interest

The authors declare that the research was conducted in the absence of any commercial or financial relationships that could be construed as a potential conflict of interest.

## Publisher’s note

All claims expressed in this article are solely those of the authors and do not necessarily represent those of their affiliated organizations, or those of the publisher, the editors and the reviewers. Any product that may be evaluated in this article, or claim that may be made by its manufacturer, is not guaranteed or endorsed by the publisher.
